# Calcium bicarbonate as an antimicrobial, antiviral, and prion-inhibiting agent (Review)

**DOI:** 10.3892/br.2022.1540

**Published:** 2022-05-12

**Authors:** Takashi Onodera, Akikazu Sakudo, Yoshifumi Iwamaru, Takashi Yokoyama, Makoto Haritani, Katsuaki Sugiura, Hidekatsu Shimakura, Takeshi Haga, Rumiko Onishi, Koichi Furusaki

**Affiliations:** 1 Laboratory of Environmental Science for Sustainable Development, Department of Veterinary Medical Sciences, The University of Tokyo, Tokyo 113-8657, Japan; 2 Research Center for Food Safety, Graduate School of Agricultural and Life Sciences, The University of Tokyo, Tokyo 113-8657, Japan; 3 School of Veterinary Medicine, Okayama University of Science, Imabari, Ehime 794-0051, Japan; 4 National Institute of Animal Health, Tsukuba, Ibaraki 305-1002, Japan; 5 Division of Infection Control and Disease Prevention, Department of Veterinary Medical Sciences, Graduate School of Agricultural and Life Sciences, The University of Tokyo, Tokyo 113-8657, Japan; 6 Santa Mineral Co., Ltd., Tokyo 105-0013, Japan; 7 Mineral Activation Technical Research Center, Omuta, Fukuoka 836-0041, Japan

**Keywords:** calcium carbonate, human norovirus, mesoscopic structure, prions, SARS-CoV-2, *Xanthomonas campestris* pv. *campestris*

## Abstract

Calcium bicarbonate does not act as a disinfectant at neutral pH; however, it exerts strong antimicrobial activity after it is placed in a high-voltage electric field, whereby it assumes an alkaline pH (12.4). Moreover, the microbicidal activity of the resulting solution (named CAC-717) is not influenced by the presence of organic material or resistance of the agent to inactivation. When sprayed on the skin surface, the pH of CAC-717 decreases rapidly to 8.84. CAC-717 comprises fine particles of 50-500 nm. When these mesoscopic crystals are dissolved in water, they destroy the genomes of bacteria or viruses and neutralize the infectious properties of abnormal prion proteins produced in ScN2a cells. The severe acute respiratory syndrome coronavirus type 2 (SARS-CoV-2) pandemic has resulted in unprecedented international demand for disinfectants. A small titer of SARS-CoV-2 remains infectious even after 30 sec in growth medium at pH 12.4. CAC-717 has exhibited a strong virucidal effect (3.6 to 4.4 log_10_ decrease) against all examined SARS-CoV-2 isolates, including mutant forms. Similarly, human noroviruses also remain intact at pH 12.4; however, CAC-717 has been shown to cause a 3.25 log_10_ reduction in norovirus genomic RNA compared to untreated samples. Existing evidence suggests that an unidentified mechanism controls the virucidal activity of CAC-717.

## 1. Introduction

Sodium bicarbonate and potassium bicarbonate exert microbicidal effects against plant bacteria and fungi under alkaline conditions. In nature, these carbonate solutions dissolve quickly, making them effective against specific pathogens and small insects. In contrast, calcium bicarbonate solutions at pH 7.0 do not exhibit microbicidal activity against bacteria or fungi. However, when high-voltage electricity is applied to a calcium bicarbonate solution, mesoscopic crystals (50-500 nm in size) are formed (1). At pH 12.4, these crystals, named CAC-717, exert multiple microbicidal effects against animal and plant pathogens, allowing them to be characterized as ‘soft’ agricultural chemicals. In another recent study, the authors reported that CAC-717 efficiently inactivated a variety of infectious agents, including prions (2).

In the context of the ongoing coronavirus disease 19 (COVID-19) pandemic, the World Health Organization recommends ‘to ensure that environmental cleaning and disinfection procedures are followed consistently and correctly. Thoroughly cleaning environmental surfaces with water and detergent and applying commonly used hospital-level disinfectants, such as sodium hypochlorite, are effective and sufficient procedures’ (3). Although some antiseptics/disinfectants, including ethanol and sodium hypochlorite, exhibit significant activity against the recently emerged severe acute respiratory syndrome coronavirus type 2 (SARS-CoV-2), they are harmful to human cells and must be used at elevated concentrations (4-7). Furthermore, the presence of organic material significantly reduces the activity of chlorine-derived compounds (6). In the present review, the effect of CAC-717 as an environmentally friendly disinfectant against emerging and recurrent infectious pathogens was described.

## 2. Calcium bicarbonates

### Effects of calcium bicarbonates

Calcium is a major macronutrient for trees and is used in agricultural fertilizers. It is important for preserving membrane and cell wall integrity, as well as intracellular transport (8). At pH 7, calcium bicarbonate lacks a mesoscopic structure and does not exert a cytotoxic effect against microorganisms.

Calcium bicarbonate, whose chemical formula is Ca(HCO_3_)_2_, does not refer to an actual solid compound, as it exists only in dilute aqueous solutions containing calcium (Ca^2+^), bicarbonate (HCO_3_^-^), and carbonate (CO_3_)^2-^ ions together with dissolved carbon dioxide (CO_2_). The relative concentration of these carbon-containing species depends on the pH, with bicarbonate being predominant at pH 6.36-10.25 in water. These materials are often used in chewing gums with protective action on the oral cavity (9). In clinical practice, calcium bicarbonate water is used to treat patients with gastroesophageal reflux disease (10).

Compared to untreated specimens, CAC-717 treatment has been shown to reduce human norovirus genomic RNA by 3.25 log_10_ (11). The virucidal effect of CAC-717 was compared to that of a phosphate buffer control at pH 12.4 and calcium bicarbonate without the mesoscopic structure (pH 7.0) (11). A human norovirus (GII.4 Sydney 2012) (12) was used as a target and was purified prior to disinfectant treatment. Phosphate buffer at pH 12.4 and calcium bicarbonate at pH 7.0 failed to inactivate human norovirus (11). Although CAC-717 is an alkaline solution, its pH decreases rapidly (within 1 min) to 8.84 upon contact with human skin (1). Indeed, CAC-717 is classified as non-irritant (Class 0) and skin sensitization tests conducted in rabbits according to the Ministry of Health, Labour and Welfare of Japan Guidelines, Biological Evaluation of Medical Devices-Part 10: Test for irritation and skin sensitization (ISO 10993-10, July 2, 2006) have not revealed any harmful properties (1). Furthermore, rabbit eye toxicity tests using the OECD Guidelines for Testing Chemicals no. 405: Acute Eye Irritation/Corrosion, performed under the same animal welfare requirements (ISO 10993-2, July 2, 2006) confirmed CAC-717 biosafety (1). Concentrated CAC-717 exhibited cytotoxicity in cultivated cell lines (11); but even this effect was lost after a 1:2 dilution in phosphate buffer.

### Preparation of calcium bicarbonate with a mesoscopic structure

**Figure 1:**
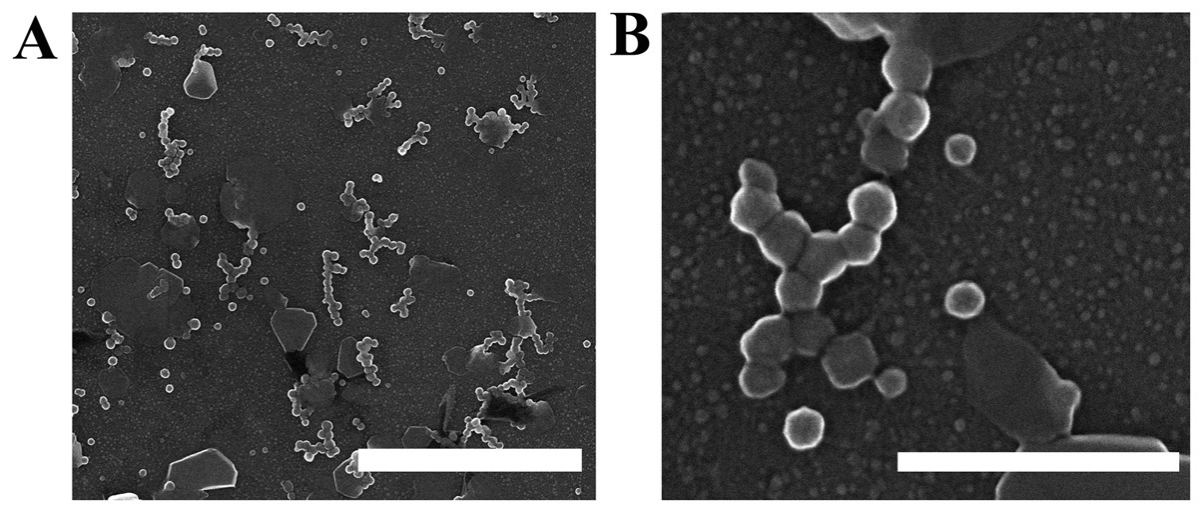
Scanning electron microscopy images of mesoscopic CAC-717 particles generated after high-voltage treatment of a calcium bicarbonate aqueous solution. The images were obtained using a Hitachi S-4800 electron microscope at 15 kV with a (A) magnification of x20,000 and scale bar, 2000 nm and (B) magnification of x100,000 and scale bar 500 nm.

**Figure 2:**
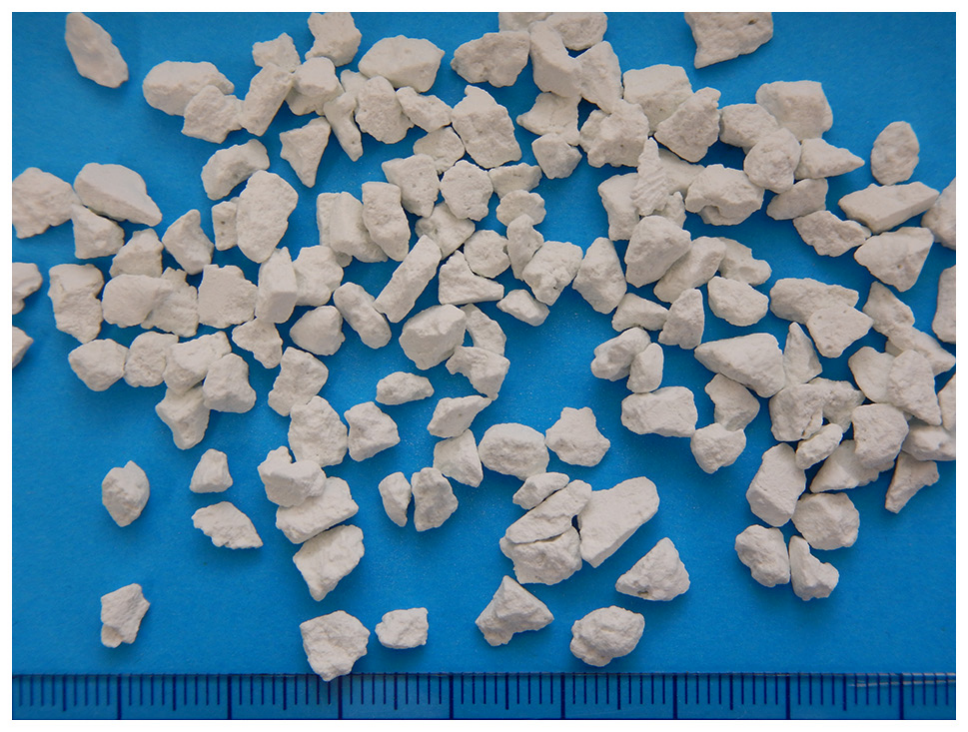
CAC-717 stones. A scale ruler is shown below (each small division is 1 mm).

To obtain CAC-717, an electric field is applied to mineral water containing calcium bicarbonate (1,2,13,14). According to the Japanese patent No. 5778328, CAC-717 (Food and Drug Administration/USA Regulation no. 880.6890 Class 1 disinfectant) is produced by applying a voltage of 2x10^4^ V for 48 h using Teflon-coated electrostatic-field electrodes (2). The resulting material has a pH of approximately 12.4 and contains 6.9 mM calcium bicarbonate particles (81,120 mg/l) with a mesoscopic structure (50-500 nm) that can be observed under an electron microscope (Fig. 1). CAC-717 is adsorbed onto a ceramic surface and air-dried for storage in the form of CAC-717 stones (Fig. 2). Prior to use, the CAC-717 stones are placed in fresh distilled water, calcium bicarbonate is dissolved, and CAC-717 is reconstituted. Reconstituted CAC-717 has the same microbicidal properties as original CAC-717.

The CAC-717 suspension, which is a colorless disinfectant, can be sprayed and dried on metal or plastic surfaces, resulting in a white powder coating (1). Scanning electron microscopy samples were obtained from a CAC-717 suspension after drying on a glass slide and used for quality control assessment, together with pH and calcium bicarbonate content measurements (1).

## 3. Effect of mesoscopic calcium bicarbonate particles on animal and plant pathogens

### Bacteria

**Figure 3:**
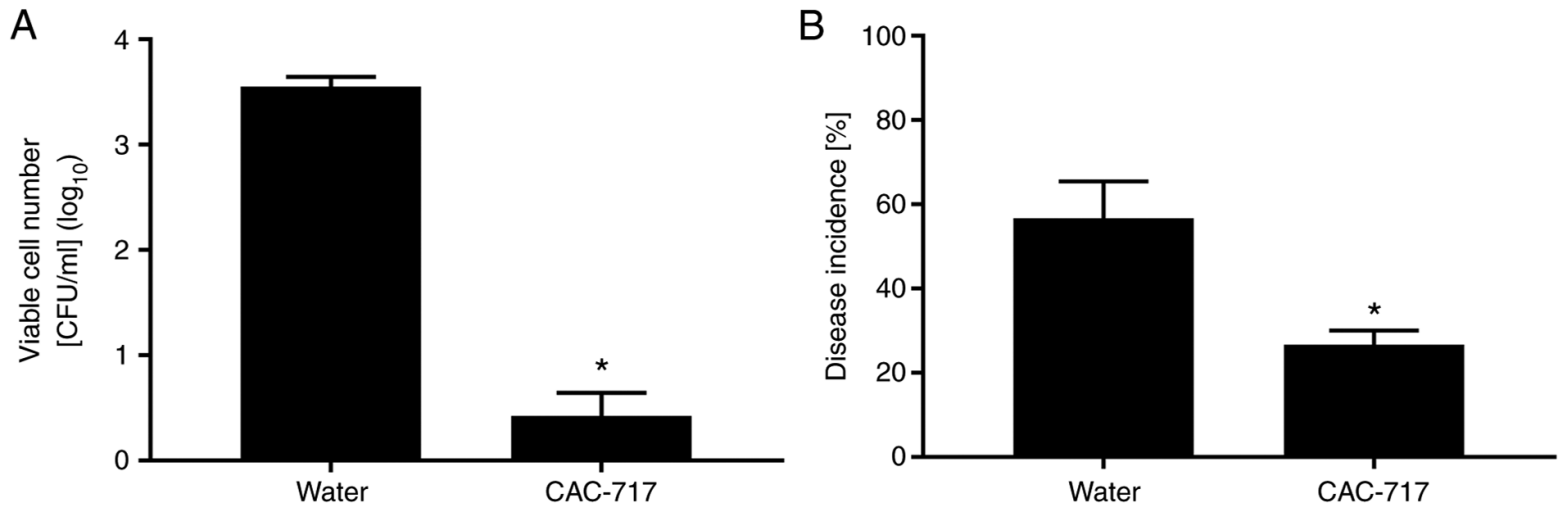
CAC-717 treatment of cabbage seeds contaminated with *Xcc*. Seeds were treated with distilled water or CAC-717 at 25˚C for 30 min. (A) Viable cell counts (expressed as colony-forming units) of *Xcc* recovered from seeds and incubated at 25˚C on yeast extract dextrose CaCO_3_ medium for 3 days. (B) Incidence of disease in CAC-717-treated or untreated seeds. Data are derived from triplicate samples and are representative of the mean ± standard error of the mean from two experiments. ^*^P<0.05 (Mann-Whitney U test). Cited from Sakudo *et al* (14) under the terms of the Created Commons Attribution 4.0 International license. *Xcc*, *Xanthomonas campestris* pv. *campestris.*

CAC-717 has been shown to be effective in inactivating plant pathogens (14). Black rot is a disease affecting cabbage and other cruciferous plant leaves, and is caused by *Xanthomonas campestris* pv. *campestris* (*Xcc*), a gram-negative seed-borne bacterium (15). The number of viable *Xcc* cells after treatment with CAC-717 was determined. An *Xcc* suspension (8.22 log_10_ colony-forming units/ml) was incubated with an equal volume of CAC-717 at 25˚C for different periods of time (Table I). CAC-717 treatment caused a decrease in the number of *Xcc* cells after 0.5 min (Fig. 3A). In control experiments, no significant reduction in the number of *Xcc* cells was observed after treatment with distilled water at 25˚C; whereas hot water (50˚C) treatment significantly decreased the number of *Xcc* cells (14). In test samples, CAC-717 caused a low incidence of black rot, with only 26.77±3.33% of seeds exhibiting signs of disease after incubation at 25˚C for 5 days, compared with 56.67±8.82% in the distilled water group (Fig. 3B). No significant difference in the germination rate and plant stem length was detected between distilled water (25˚C) and CAC-717 treatment after cultivation for 5 days.

CAC-717 displays a bactericidal effect against *Escherichia coli* (*E. coli*) and *Salmonella enterica* (*S. enterica*) (13) (Table I). Following treatment with CAC-717 for 2 min, viable *E. coli* cells decreased by approximately 3.00-2.00 log_10_ and *S. enterica* by more than 7.00 log_10_, indicating that some bacteria are more sensitive than others to CAC-717. When using sodium hypochlorite (4 ppm) as a fungicide for chicken eggs mixed with *S. enterica*, the average multiple endpoint *D* value was 0.195 min (16). In contrast, CAC-717 displayed an average multiple-endpoint *D* value of 0.080 min, indicating a more powerful bactericidal effect than sodium hypochlorite.

### Prions

Extreme autoclaving conditions (134˚C, 18 min) are required to inactivate prions, although some prions retain transmissibility even after dry-heating at 400˚C (16). Alternatively, prions are inactivated upon treatment with sodium dodecyl sulfate, sodium hydroxide, and sodium hypochlorite (17), but these chemicals are generally impractical due to their corrosive properties.

**Figure 4:**
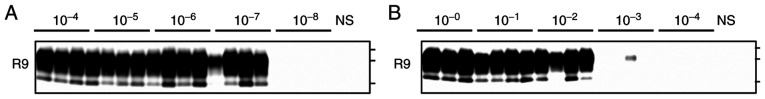
PrP^Sc^ inactivation evaluated by PMCA. Brain homogenate of CD-1 mice was used as PrP^Sc^ source to infect N2a cells, and PMCA buffer was used to dilute samples (dilution series are indicated above the blots). PMCA products from R9 of amplification were analyzed by western blotting after proteinase K digestion (2). (A) Control samples treated with PBS. (B) CAC-717-treated samples. Except for the NS, amplification was performed in quadruplicate. Molecular mass markers are indicated on the right-hand side. Cited from Sakudo *et al* (2) under the Created Commons Attribution 4.0 International license. PrP^Sc^, scrapie prion; PMCA, protein misfolding cyclic amplification; R9, round 9; NS, non-seeded control.

In another recent study, the authors reported that CAC-717 inactivated prions (2) (Table I). Western blotting was used for scrapie prion (PrP^Sc^) analysis (18). To examine the seeding activity of PrP^Sc^, protein misfolding cyclic amplification (PMCA) was used, which mimics the *in vivo* reaction and amplifies PrP^Sc^ *in vitro* (19). Western blotting and PMCA were applied to follow proteinase K-resistant PrP^Sc^, while a transgenic mouse bioassay was used to test the transmissibility of prions. Western blot analysis showed that the levels of proteinase K-resistant PrP^Sc^ in lysates obtained from prion-infected N2a cells were markedly reduced after CAC-717 treatment. Next, PMCA was used to assess the conversion activity of any remaining PrP^Sc^ after CAC-717 treatment. The seeding activity of PrP^Sc^ was also clearly reduced in CAC-717-treated samples (2) (Table I). In addition, mice injected with CAC-717-treated samples survived longer than those injected with PBS-treated controls. These findings suggest that CAC-717 reduced both PrP^Sc^ conversion activity and prion transmissibility (Fig. 4).

Further studies are required to determine the mechanism by which CAC-717 inactivates prions. Given that CAC-717 does not cause irritation and is not corrosive, it offers an alternative to other prion disinfectants such as hypochlorous acid (20); however, detailed comparison with the latter is still needed.

### Animal viruses

Recently, CAC-717 was tested against SARS-CoV-2(21). CAC-717 exhibited a strong virucidal effect against all the examined mutant forms of SARS-CoV-2 isolated in Japan. Viral infectivity decreased by 3.8 to 4.2 log_10_ within 15 sec (Table II), implying a virucidal activity similar to that of 80% ethanol. This strong microbicidal effect of CAC-717 has been linked to its elevated alkalinity, which may be due to the relative decrease in H^+^ and concomitant increase in OH^-^. However, given that SARS-CoV-2 retains its infectivity for up to 30 sec at pH 12.4 (pH-adjusted Dulbecco's modified Eagle's medium) (21), it is more likely that an unidentified mechanism controls the virucidal activity of CAC-717. SARS-CoV-2 is known for the emergence of numerous strains. In addition to the conventional original virus (WK-521), the virucidal activity of CAC-717 was confirmed against α, β, γ, and δ variants, with the log_10_ reduction in infectivity ranging from 3.6 to 4.4 (Table II). The effectiveness of CAC-717 in the presence of organic matter was analyzed to confirm its beneficial properties in the general environment (21). The tissue culture infectious dose of the virus mixed with 5% bovine serum albumin was 10^4.8^ and the virucidal effect of CAC-717 corresponded to >4.3 log_10_ (21). A lower virucidal effect of sodium hypochlorite has been observed in the presence of organic material; whereas the value obtained for CAC-717 indicates only a minor reduction. Numerous disinfectants have been used for COVID-19 control; hence, their impact on human health and the environment should be taken into account. As CAC-717 is harmless and does not cause skin or eye toxicity in rabbits (1), its future use as an anti-COVID-19 disinfectant is less problematic. Moreover, because the calcium bicarbonate component of CAC-717 is derived from plant material, it is non-flammable and can be used in a variety of applications for which ethanol is not suitable (21).

In addition to SARS-CoV-2, a recent study showed that CAC-717 possessed virucidal activity against 22 different types of DNA or RNA viruses. Destruction of viral nucleic acids in RNA viruses has been observed (22), but the exact effect of CAC-717 against viral DNA remains to be determined (22). In nature, human norovirus is typically associated with the fecal matrix. To investigate the microbiological properties of CAC-717, four fecal specimens were evaluated. Compared to the untreated specimen, CAC-717 resulted in log_10_ reductions of 1.36, 2.78, 1.64, and 3.52 in the GI.2, GII.4 Sydney 2012, GII.5, and GII.7 specimens, respectively (Table I). Thus, CAC-717 could successfully inactivate human norovirus under clinical conditions. A similar effect was reported with heat treatment, but not with 1,000-ppm sodium hypochlorite in a stool suspension (except for human norovirus GII.17) (11) or 70% (v/v) ethanol.

CAC-717 has been proven effective against influenza virus (1) and feline calicivirus (FCV) (19) (Table I). To investigate the mechanistic effect of CAC-717 against viruses, real-time PCR revealed a progressive decline in the integrity of the FCV genome with increasing CAC-717 treatment time, as opposed to only intact RNA from untreated FCV samples (13).

Hypochlorous acid solution is also effective against several viruses (23,24); however, it is less stable in the presence of ultraviolet irradiation or in contact with air or organic compounds. Consequently, hypochlorous acid must be stored under cool and dark conditions to maintain its microbicidal activity (23).

Recently, concentrated bioshell calcium oxide (BiSCaO) water has been reported as an effective skin disinfectant (7,25). Although BiSCaO is strongly alkaline (pH 12.8), the pH of BiSCaO water decreased to 8.5 within 5 min after spraying on the back skin of hairless rats. The generation of CaCO_3_ following the interaction between Ca^2+^ ions in BiSCaO water and CO_2_ in air was suspected to be the cause of this rapid drop in pH (25). This finding echoes the rapid decrease in pH of CAC-717 when in contact with rabbit skin (1).

## 4. Conclusion

The SARS-CoV-2 pandemic has caused both a massive healthcare crisis and enormous economic damage worldwide. With increasing hygiene and safety challenges, disinfection with electrolyzed water is of potential significance in the clinical field as a means to cut off the route of viral transmission (21,26). CAC-717 has strong antimicrobial efficacy without associated irritation (21), highlighting potential applications in the food and medical sectors for disinfecting hard surfaces, in addition to its established anti-parasitic role in agriculture.

After the discovery that pangolins could harbor SARS-CoV-2-related coronaviruses, Chinese authorities banned the trade and sale of these animals. Currently, it is too early to evaluate the overall impact of SARS-CoV-2 on wild animal management and conservation. A recent study by a Japanese group identified a bat sarbecovirus loosely related to SARS-CoV-2(27). Future research efforts should focus on the likely origins of novel pathogens, along with the development of vaccines and drugs (28,29). SARS-CoV-2 RNA was detected in secondary-treated wastewater samples when COVID-19 cases peaked in the community (30). The present study suggests that, when dried CAC-717 stones are placed in fresh water, calcium bicarbonate is dissolved and CAC-717 is reconstituted. Existing evidence suggests that reconstituted CAC-717 retains the same microbicidal activity as original CAC-717. These CAC-717 stones may be useful for eliminating SARS-CoV-2 from the environment, including sewage and running water. Daily cleaning and disinfection of surfaces in hospitals are essential to limit the spread of infection (31). The US Environmental Protection Agency recommends the use of disinfectants with hypochlorite acid as the active ingredient for disinfection of surfaces to combat SARS-CoV-2(31); however the mild toxicity of this agent calls for the use of alternatives such as CAC-717.

This review highlights the latest developments and new perspectives related to CAC-717, especially its application in clinical fields. In the future, CAC-717 may be applied on the surface of numerous materials as a safe and environmentally friendly antiseptic/disinfectant. Further studies and validation in other model systems are required to better understand its mechanism of action and untapped potential.

## Figures and Tables

**Table I:** Antimicrobial, antiviral, and prion-inhibiting effects of CAC-717.

		Pathogen titer		
Pathogen	Duration of treatment (min)	Untreated	CAC-717	(Refs.)
*Xanthomonas campestris* pv. *campestris*	0.5	8.22 log_10_ CFU/ml	5.63 log_10_ CFU/ml	(15)
*Escherichia coli*	2	1.52x10^9^±0.35x10^9^ CFU/ml	7.50x10^6^±2.50x10^6^ CFU/ml	(14)
*Salmonella enterica*	2	2.14x10^7^±0.12x10^7^ CFU/ml	Undetectable	(14)
*Murine norovirus*	1	5.98 log_10_ TCID_50_/ml	Undetectable	(12)
*Feline calicivirus*	2	7.26x10^5^±2.70x10^5^ TCID_50_/ml	<10 TCID_50_/ml	(14)
*Influenza virus*	15	6.58 log_10_ TCID_50_/ml	<1.50 log_10_ TCID_50_/ml	(1)
*Scrapie prion*	60	9.95 log_10_ PMCA_50_/ml	5.20 log_10_ PMCA_50_/ml	(2)

Detailed materials and methods are reported in each reference. CFU, colony-forming unit; PMCA, protein misfolding cyclic amplification; TCID_50_, tissue culture infectious dose.

**Table II:** Virucidal efficacy of CAC-717 against SARS-CoV-2.

		Viral titer (TCID_50_/ml)	
Strain	Variant type	Distilled water	CAC-717
SARS-CoV-2/WK-521	Original	4.9	≤0.6
SARS-CoV-2/KH-1/2021	Original	5.0	≤0.6
hCoV-19/Japan/QK002/2020	α	4.2	≤0.6
hCoV-19/Japan/TY8-612/2021	β	4.8	≤0.6
hCoV-19/Japan/TY7-501/2021	γ	4.4	≤0.7
SARS-CoV-2/KH-25/2021	δ	4.9	≤0.6

Aliquots of virus were mixed with 49 volumes of CAC-717, incubated for 5 min, and SARS-CoV-2 titers were measured (22). TCID_50_, tissue culture infectious dose.

## Data Availability

Not applicable.
